# Enzymatic Hydrolysis of a Collagen Hydrolysate Enhances Postprandial Absorption Rate—A Randomized Controlled Trial

**DOI:** 10.3390/nu11051064

**Published:** 2019-05-13

**Authors:** Kathrine Skov, Mikkel Oxfeldt, Rebekka Thøgersen, Mette Hansen, Hanne Christine Bertram

**Affiliations:** 1 Department of Food Science, Aarhus University, Kirstinebjergvej 10, DK-5792 Aarslev, Denmark; 20095682@post.au.dk (K.S.); rebekka.thoegersen@food.au.dk (R.T.); 2 Section for Sport Science, Department of Public Health, Aarhus University, Dalgas Avenue 4, 8000 Aarhus, Denmark; Mikkeloxfeldt@hotmail.com (M.O.); mhan@ph.au.dk (M.H.)

**Keywords:** collagen absorption, glycine, proline, hydroxyproline, nutrimetabolomics, collagen nutriceuticals, sports nutrition, amino acid absorption, dietary protein, collagen uptake

## Abstract

Collagen is characterized by its high content of glycine, proline and hydroxyproline, and is found to exert beneficial effects on joint pain related to activity and osteoarthritis. However, to exert any beneficial effects it is essential that collagen is optimally absorbed. This study aimed to investigate the postprandial absorption of collagen and elucidate the impact of an exogenous enzymatic hydrolysis on absorption rate and bioavailability. A randomized, blinded, cross-over study was conducted where ten healthy male subjects received either 35 g enzymatically hydrolyzed collagen protein (EHC), 35 g non-enzymatically hydrolyzed collagen protein (NC) or placebo (250 mL water) on three nonconsecutive days. Blood samples were drawn before, and up to 240 min following, ingestion and the blood metabolome was characterized by nuclear magnetic resonance (NMR)-based metabolomics. A significant increase in the plasma concentration of nearly all amino acids (AAs) was observed over a 240 min period for both EHC and NC. In addition, the absorption rate and bioavailability of glycine, proline and hydroxyproline were significantly higher for EHC (*p* < 0.05). In conclusion, ingestion of collagen hydrolysates increases postprandial plasma concentrations of AAs over a period of 240 min, and an enzymatic hydrolysis increases the absorption rate and bioavailability of the collagen-rich AAs glycine, proline and hydroxyproline.

## 1. Introduction

Chronic tendinopathy, ligament ruptures, and bone fractures are common injuries among athletes. The consequences can be severe for elite athletes, who may be forced to end their career, since treatment in a majority of patients is incomplete and unsuccessful from the perspective of a return to play at the elite level [1,2]. Furthermore, osteoarthritis (OA) is a chronic condition affecting the joints, and the progressive degeneration of articular cartilage causes pain and functional disability. It is estimated that more than 250 million people are affected by OA worldwide and to this day no curative treatments are available [3]. Therefore, there is an obvious need to search for alternative treatment strategies to optimize rehabilitation. Bones, tendons, ligaments, and cartilage are constituted by a complex matrix but are characterized by a high content of collagen, and this structural protein is vital for the structure and biomechanical properties of these musculoskeletal tissues [4]. Collagen is an abundant structural protein present in connective tissue. Besides its nutritional value as a protein source, dietary supplementation with collagen-derived peptide sources has been suggested to provide beneficial effects in patients with tendinopathy [5,6,7], chronic joint instability [8], osteoarthritis (OA) [3,9,10,11,12], and activity-related joint pain [13,14]. Thus, nutritional interventions focusing on increasing the amino acid (AA) components of collagen have been suggested to improve collagen synthesis of collagen-rich tissues such as ligaments and bones [5] and potentially slow the degenerative process in OA affected joints [11]. However, in order to exert any potential beneficial effects, optimal digestion and absorption of AA components of collagen is pivotal. Protein digestibility generally varies depending on dietary source and processing methods [15]. Enzymatic hydrolysis of proteins is expected to influence protein digestion and the postprandial plasma AA profile, and previous studies on milk proteins have shown an enhanced postprandial AA absorption and bioavailability when the protein source was subjected to an enzymatic hydrolysis [16]. Congruently, it is expected that an enhanced absorption rate will be observable when the collagen protein structure is manipulated through an enzymatic hydrolysis. In nutrition science, metabolomics provides a characterization of the wide-ranging scale of metabolites (the metabolome) and enables a comprehensive comparison of the metabolic responses after different dietary interventions [17]. ^1^H NMR spectroscopy is able to detect any mobile proton-containing metabolites with a low-molecular weight [17], and ^1^H NMR-based metabolomics spectroscopy has recently been shown to be a valuable tool for the characterization of postprandial blood plasma AA profiles [18,19]. Thus, ^1^H NMR-based metabolomics applied to blood plasma samples provides a framework for elucidating the effect of an enzymatic hydrolysis on the bioavailability and absorption of collagen. To the best of our knowledge, investigations on the effects of enzymatic hydrolysis of collagen protein on the postprandial absorption rate have not been reported yet. Consequently, the aim of the present study was to examine the postprandial absorption of collagen hydrolysates and to elucidate the impact of an exogenous enzymatic hydrolysis on the absorption rate and bioavailability. For this purpose, an acute human intervention study with a cross-over design was conducted with two collagen-containing products; an enzymatically hydrolyzed collagen protein (EHC), and a non-enzymatically hydrolyzed collagen protein (NC), and the blood plasma metabolome was characterized by ^1^H NMR-based metabolomics. We hypothesized that the increase in circulating amino acids would be faster and more pronounced after ingestion of EHC compared to NC.

## 2. Materials and Methods 

### 2.1. Subjects

Ten healthy young males were recruited from the Section of Sports Science, Department of Public Health at Aarhus University. The recruitment was initiated on 8 November 2018 and the last experimental day including subjects was completed on 15 December 2018. Participants were healthy males in the age range 18–35. Participant characteristics are provided in Table 1. Exclusion criteria included: the use of medication that could influence postprandial absorption; muscular, tendon or metabolic illnesses; smoking; hypertension; use of supplements during the trial period; weight loss or gain of more than 2 kg during the trial period; and working night shifts. The trial complied with the Declaration of Helsinki and was approved by The Central Denmark Region Committees on Health Research Ethics (Journal 65141, case number 1-10-72-287-18). Furthermore, the study was registered at ClinicalTrials gov (NCT03749239) and adhered to the CONSORT guidelines. All participants gave their informed consent before the intervention was carried out. 

### 2.2. Design and Experimental Protocol

**Figure 1:**
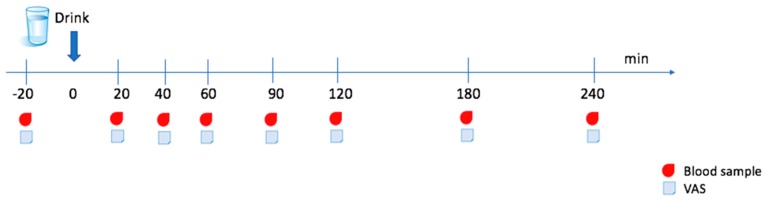
Experimental protocol and fixed time points for blood sample collection and visual analogue scale (VAS) questionnaire. Time of drink consumption was designated as time zero (0 min).

The study was a randomized, blinded, placebo-controlled, cross-over study consisting of three nonconsecutive experimental days at the Section for Sports Science, Department of Public Health at Aarhus University. On the experimental days, participants consumed a collagen supplement of 35 g crude protein enzymatically hydrolyzed collagen (EHC), or non-enzymatically hydrolyzed collagen (NC), or placebo (water) in restricted random order based on the recruitment order to ensure that an equal number of subjects received the three intervention beverages on experimental days one, two and three, respectively. One of the primary investigators (MH), who did not take an active part in the blood sampling procedures or the following laboratory analysis, performed the randomization. The subjects were blind to the content and order of the intervention beverages. Furthermore, the laboratory staff were not informed about the sample id for the intervention beverages until the statistical analysis was initiated. Participants were instructed to record their dietary intake and physical activity level on the day before the first trial. These recordings were used as a standard plan before the following trial days to ensure standardization of dietary intake and physical activity level. On trial days, participants arrived in the morning after an overnight fast and consumed a drink containing either EHC, NC, or placebo dissolved in 250 mL water. The drinks were consumed within 2 min and the time was designated as time zero. Immediately following consumption of the drink, all participants consumed 100 mL water to attenuate the mouthfeel and taste of the trial product. Additional blood samples were drawn at fixed time points for four hours following beverage consumption (Figure 1). Furthermore, participants were instructed to answer three questions on a visual analogue scale (VAS) after every blood sample collection. All participants remained in a resting position and were not allowed to eat or drink throughout the trial hours. There were at least two days between the experimental days. The primary outcome measure was the time to peak amino acid concentration and the accumulated amino acid concentration of the individual amino acids after ingestion of the intervention beverages.

### 2.3. Collagen Products

The collagen supplements were manufactured and provided by Essentia Protein Solutions A/S, Denmark. Two different types of collagen products were included: an enzymatically hydrolyzed collagen (EHC) (OmniCol^TM^ 110, Essentia Protein Solutions, Graasten, Denmark) and a non-enzymatically hydrolyzed collagen-containing product (NC) (HydroBEEF^TM^, Essentia Protein Solutions, Graasten, Denmark). Both products were manufactured from beef bone, and the collagen products did not contain any additives, sweeteners or flavors. OmniCol^TM^ 100 and HydroBEEF^TM^ are commercial products and thus approved for human consumption according to GRAS. Accordingly, the products’ content of heavy metals has been analyzed and the values are considered safe. We have not conducted any further testing of the products in relation to safety, but a recent study with a similar collagen product revealed that the product did not elicit any toxic effects in an in vitro model [20].

Nutritional contents and amino acid profiles are provided as supplementary material. Collagen powder corresponding to 35 g protein was dissolved in 200 mL ~50 °C hot water. Prior to ingestion, four ice cubes (~50 mL) were added to allow drinks to cool. 

### 2.4. Blood Samples

In each experimental trial, eight blood samples of ~8 mL were collected into evacuated containers containing lithium heparin. The blood samples were collected 20 min before ingestion of the intervention beverage and at 20, 40, 60, 90, 120, 180 and 240 min after ingestion of the intervention beverage (Figure 1). The blood samples were centrifuged at 1200× *g* for 10 min at 4 °C to separate the plasma and stored at −80 °C until further analysis.

### 2.5. Visual Analogue Scale (VAS)

Sensation of hunger, satiety and fullness were measured using a VAS, consisting of three questions. The VAS consisted of a 73 mm horizontal unbroken line with a statement anchored at each end describing the extremes, e.g., “I am not hungry at all”, “I have never been more hungry”, “I am not full at all”, “I am very full”. Each set of VAS questions was answered at every blood sampling. The intensity of the feeling (distance of the vertical mark from the origin on the left) was measured, yielding a score in the range 0–73 mm. 

### 2.6. H NMR Spectroscopy 

Thawed plasma samples were filtered using 10K Amicon Ultra centrifugal filter units (Merck Milipore Ltd., Cork, Ireland). Prior to use, the filters were washed four times with 500 μL MilliQ water to remove traces of glycerol. After washing, 500 μL plasma was added to the filters and centrifuged at 4 °C, 14,000× *g* for 2 h. Subsequently, 400 μL plasma filtrate was transferred to a 5 mm NMR tube containing 100 μL sodium-hydrogen phosphate buffer (50 mM Na_2_HPO_4_, pH 7.4), 25 μL deuterium oxide (D_2_O) with 0.05% TSP and 75 μL D_2_O. ^1^H NMR spectroscopy was performed on a Bruker Avance III 600 MHz NMR spectrometer operating at a ^1^H frequency of 600.13 MHz with a 5 mm ^1^H TXI probe (Bruker Biospin, Reinstetten, Germany). Spectra were acquired at 298 K using the one-dimensional (1D) nuclear Overhauser enhancement spectroscopy (NOESY)-pulse sequence presat for water suppression (NOESYPR1D). The acquisition parameters used were: 64 scans (NS), spectral width (SW) = 7288 Hz (12.1450 ppm), data points (TD) = 32,768, relaxation delay (D1) = 5.0 s and acquisition time (AQ) = 2.25 s. The free induction decays (FIDs) were multiplied by a line-broadening function of 0.3 Hz prior to Fourier transformation. A total of 263 samples were analyzed, and the spectra obtained were baseline and phase corrected using TopSpin 3.0 (Bruker BioSpin, Rheinstetten, Germany). Metabolite assignment and quantification were conducted using Chenomx NMR Suite 8.13 (Chenomx Inc., Edmonton, AB, Canada).

### 2.7. Statistical Analyses

Statistical analysis and graph design were performed using GraphPad Prism (GraphPad Software Inc., San Diego, CA, USA). Blood sample concentrations and area under the curve (AUC) data were analyzed using a two-factor RM ANOVA with statistical significance defined as *p* < 0.05. All data sets were tested for normal distribution and sphericity prior to the ANOVA analysis. Normal distribution was tested using the Shapiro-Wilk test. Data were transformed by the natural logarithm if deviations from the normal distribution were observed. Sphericity was tested using Mauchly’s Test of Sphericity and a Greenhouse–Geisser correction was used if Mauchly’s Test of Sphericity showed a *p*-value of less than 0.05.

Previous trials of 35 g hydrolyzed vs. non-hydrolyzed milk protein tests have shown a difference in amino acid uptake of 1.4 mmol * 6 h/L (this difference was only evident over the first 4 h) [16]. Assuming that the difference in amino acid concentration accumulated over four hours after ingestion of EHC and NC is of a similar magnitude, a test population of 8 would enable detection of a significant difference (significance level 0.05, 80% power, SD 1.0 mmol * 4 h/L). Nevertheless, we included 10 subjects to account for possible dropout or missing data.

## 3. Results

### 3.1. H NMR Spectroscopic Analysis

^1^H NMR spectroscopy was performed on all blood plasma samples and a total of 19 plasma metabolites were identified and quantified. These included 18 AAs and glucose (Table 2). The four AAs—aspartic acid, cysteine, tryptophan and hydroxylysine—were not quantified, as their concentration was below the detection limit of the applied ^1^H NMR spectroscopic analysis.

### 3.2. General AAs Absorption

**Figure 2:**
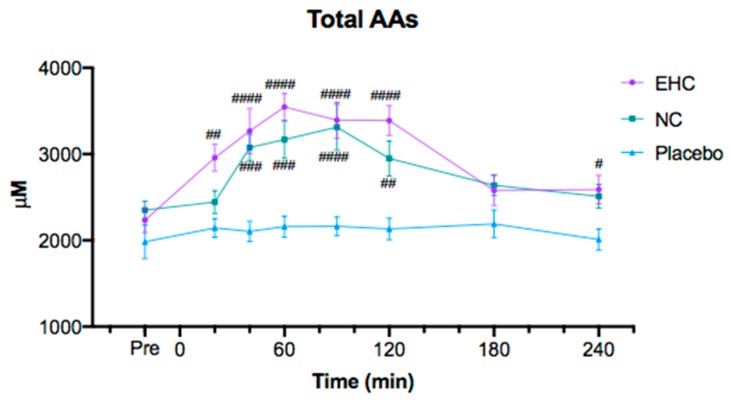
Plasma concentration of total amino acids (AAs). A significant difference was observed between enzymatically hydrolyzed collagen (EHC) and placebo as well as between non-enzymatically hydrolyzed collagen (NC) and placebo, but not between the two collagen supplements. # *p* < 0.05, ## *p* < 0.002, ### *p* < 0.0002, #### *p* < 0.0001. All data are mean ± SEM.

Figure 2 shows the total amount of AAs during the postprandial period. A significant increase in postprandial plasma concentration of total AAs was observed after ingestion of both collagen products compared to placebo. In addition, after EHC and NC intake, a significantly higher postprandial plasma concentration was observed compared with placebo (*p* < 0.05) for all AAs except histidine and methionine (Table 3). Histidine and methionine also increased after intake for both EHC and NC, however, a significant difference was only observed between NC and placebo (Table 3). For all AAs except glycine, proline, hydroxyproline and methionine, no significant difference in AUC was observed between EHC and NC. All AA concentrations peaked earlier after intake for EHC compared with NC, and for many AAs, a significant difference in AA concentration between EHC and NC was observed at 20 min after intake. Postprandial concentrations of individual AAs over time can be found in the supplementary material (Figures S1–S4).

### 3.3. Specific Collagen AAs

Overall statistical analysis showed a significant difference between EHC and NC for plasma concentrations of glycine (*p* = 0.0059), hydroxyproline (*p* = 0.0087), proline (*p* = 0.0074) and the sum of glycine, proline and hydroxyproline (Gly-Pro-Hyp) (*p* = 0.0003). In addition, plasma concentrations of these AAs were significantly different for both collagen products compared to placebo (Table 3). 

**Figure 3:**
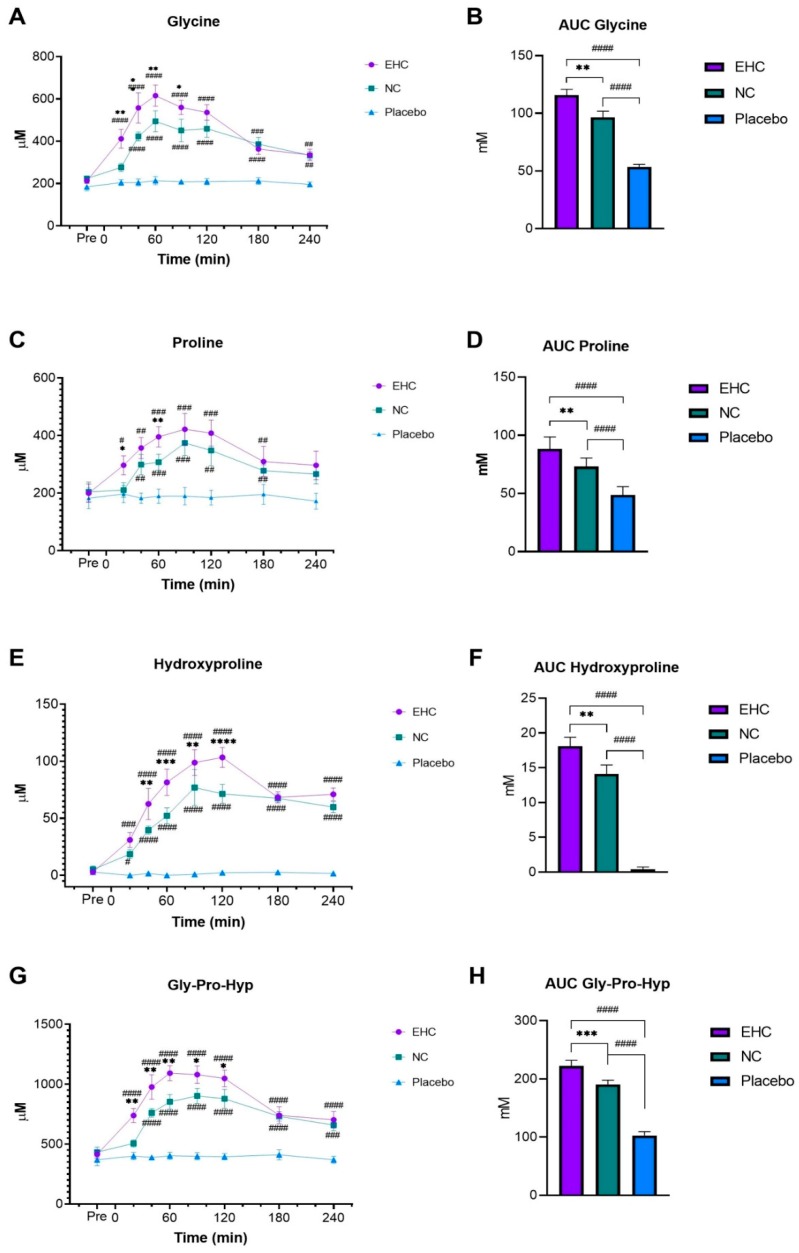
Postprandial plasma concentration of glycine (**A**), proline (**C**), hydroxyproline (**E**) and Gly-Pro-Hyp (**G**) after ingestion of enzymatically hydrolyzed collagen (EHC), non-enzymatically hydrolyzed collagen (NC) or placebo. Area under the curve (AUC) analysis for each concentration of glycine (**B**), proline (**D**), hydroxyproline (**F**) and Gly-Pro-Hyp (**H**) is evaluated from time = −20 min. * Significant difference between EHC and NC. # significant differences between either of the collagen products and placebo. * *p* < 0.05, ** *p* < 0.002 *** *p* < 0.0002, **** *p* < 0.0001, # *p* < 0.05, ## *p* < 0.002, ### *p* < 0.0002, #### *p* < 0.0001. Values are mean ± SEM.

For glycine, a significant difference was observed between EHC and NC from 20–90 min (Figure 3A). A separate analysis of the AUC showed a significant difference between EHC and NC (Figure 3B). These findings show that overall the glycine concentration was higher for EHC compared to both NC (*p* = 0.0029) and placebo (*p* < 0.0001). Plasma concentration of proline showed a significantly higher proline concentration for EHC compared to NC at 20 and 60 min (Figure 3C). A separate AUC analysis showed that overall the concentration of proline was significantly higher following consumption of EHC compared to NC (*p* = 0.0028) and placebo (*p* < 0.0001) (Figure 3D). Analysis of hydroxyproline concentrations showed a significant difference between EHC and NC collagen at all time points between 40 and 120 min (Figure 3E). Both EHC and NC showed a significantly higher concentration of hydroxyproline compared to placebo at all time points between 20 and 240 min. Furthermore, a separate AUC analysis showed an overall higher concentration of hydroxyproline following consumption of EHC compared to NC (*p* = 0.0082) and placebo (*p* < 0.0001) (Figure 3F). Analysis of the total sum of glycine, proline and hydroxyproline (Gly-Pro-Hyp) showed a significant difference between EHC and NC at all time points between 40 and 120 min (Figure 3G). In addition, both EHC and NC showed a significantly higher Gly-Pro-Hyp concentration than placebo at all time points between 20 and 240 min. A separate AUC analysis showed a significantly higher Gly-Pro-Hyp concentration following consumption of EHC compared to NC (*p* = 0.0002) and placebo (*p* < 0.001) (Figure 3H).

### 3.4. Glucose

Overall analysis of plasma glucose showed no effect of time (*p* = 0.57), treatment (*p* = 0.87) or time*treatment interaction (*p* = 0.4239). Accordingly, the AUC showed no significant effect of treatment (*p* = 0.99) or time*treatment interaction (*p* = 0.93) on the plasma concentration of glucose. Data are shown in Figure S5 in the supplementary material. 

### 3.5. VAS

Overall analysis for VAS 1 (how hungry are you) showed a significant effect of time (*p* < 0.0001), whereas no effect of treatment (*p* = 0.27) or time*treatment interaction (*p* = 0.2005) were observed. Similarly, overall analysis of VAS 2 data (how full are you) showed a significant effect of time (*p* < 0.0001), but no effect of treatment (*p* = 0.22) or time*treatment interaction (*p* = 0.65). Overall analysis of VAS 3 data (how filled up do you feel) showed a significant effect of time (*p* < 0.0001) but not of treatment (*p* = 0.275) or time*treatment interaction (*p* = 0.2203). VAS data can be found in the supplementary material (Figure S6).

## 4. Discussion

Protein hydrolysates have been suggested to be more readily digested and absorbed compared to intact proteins, thus creating a more rapid increase in plasma concentrations of AAs [16]. In the present study the postprandial responses to intake of enzymatically hydrolyzed collagen (EHC) and non-enzymatically hydrolyzed collagen (NC) were examined by ^1^H NMR-based metabolomics for the first time. AA quantification from the ^1^H NMR-based metabolomics data displayed a significantly higher plasma AA concentration following ingestion of EHC and NC compared to placebo, revealing that both products provided absorbable AAs including essential AAs. In addition, ingestion of EHC led to significantly higher plasma concentrations of glycine, proline and hydroxyproline compared to non-hydrolyzed collagen. Notably, these AAs have been connected with the potential beneficial effects of collagen supplementation on tendinopathy and articular joint pain experienced in both OA patients and athletes [7,8,9,10,11,12,13,14,21,22]. As net collagen synthesis is negative in OA patients, enhancing collagen synthesis has been proposed to help regeneration of cartilage [11,22]. Baar et al. [23] showed that AAs present in high amounts in collagen (glycine, proline, lysine, hydroxyproline and hydroxylysine) combined with vitamin C could improve collagen synthesis in a tissue-engineered ligament model. Similar findings were reported by de Paz-Lugo et al. [20], who found that glycine and proline enhanced type II collagen synthesis in bovine articular chondrocytes, suggesting that increased consumption of these AAs could be beneficial for the regeneration of the articular cartilage matrix. Oesser et al. [24] investigated the absorption of gelatin hydrolysate and its subsequent tissue distribution in mice. They found that gelatin hydrolysate accumulated in cartilage tissue, suggesting it as a potential mechanism for clinically observed benefits of gelatin consumption [24]. Whether these in vitro and animal findings can be transferred to human adults has not been established. Nevertheless, in a controlled double-blinded trial including 30 patients with mild knee OA, collagen hydrolysate supplementation seemed to have a positive effect on cartilage compared to placebo when measured by delayed gadolinium enhanced magnetic resonance imaging [11]. Furthermore, intake of 15 g of a vitamin C-enriched gelatin supplement prior to an exercise bout of 6 min rope-skipping enhanced plasma concentration of the amino-terminal propeptide of type I collagen, indicating increased collagen synthesis likely related to increase in bone type I collagen synthesis [5]. 

In the present study, the plasma concentrations of proline, glycine and hydroxyproline, following ingestion of EHC were similar to reported plasma concentrations after intake of 15 g vitamin-C-enriched gelatin supplement in the trial by Shaw et al. [5]. This finding could indicate that there is an upper threshold in regards to experiencing beneficial effect after increasing doses of collagen-derived peptides.

The concentrations of glycine, proline and hydroxyproline increased significantly at 20 min post- ingestion of EHC compared to baseline. In contrast, NC led to a significant increase in glycine, proline and hydroxyproline at 40 min post-ingestion compared to baseline. Glycine content of the EHC product was slightly higher (1.6 g/100 g, Table S1) than for the NC, whereas the content of proline and hydroxyproline was similar in EHC and NC, respectively. Consequently, the difference in the initial postprandial increase in glycine, proline and hydroxyproline concentrations, and the larger AUC for EHC compared to NC, indicate that an enzymatic hydrolysis enhances both absorption rate and bioavailability of these collagen AAs. Previous studies have investigated the absorption rate and plasma concentration of AAs following ingestion of intact proteins and their hydrolysate. However, these studies have primarily focused on whey and casein proteins and results have been conflicting. Koopman et al. [16] and Calbet et al. [25] reported an increased absorption rate of hydrolyzed casein compared to its intact protein, while Schmedes et al. (2018) found no significant difference between casein and its hydrolysate [26]. Hydroxyproline concentration was observed to peak at 120 min and 90 min post-ingestion of EHC and NC, respectively. Though hydroxyproline peaked later following EHC, it reached a higher plasma concentration. Plasma concentrations of glycine and proline peaked 60 and 90 min after intake of EHC and NC, respectively. Previous studies have reported similar findings with a peak in hydroxyproline after 120 min and a subsequent decrease to half the maximum after four hours following consumption of hydrolyzed collagen [20,27]. 

Plasma concentrations of alanine, asparagine, glutamate, glutamine, histidine, serine, threonine and valine were significantly higher over the 240 min period after consumption of both EHC and NC compared to placebo. In addition, plasma concentrations of total AA, essential AAs and branched chain amino acids (BCAAs) did not differ between EHC and NC, indicating that an enzymatic hydrolysis did not have a significant effect on bioavailability and absorption rate for these AAs. Similar findings have previously been observed for hydrolyzed casein and intact casein [26]. Plasma concentrations of arginine, leucine, lysine, tyrosine, isoleucine and phenylalanine increased significantly from 20 to 60 min following ingestion of EHC and NC, with no significant differences between the two products. Plasma concentrations of alanine, asparagine, glutamate, glutamine, histidine, serine, threonine and valine showed an increased absorption from 20 to 120 min, after which levels decreased back to baseline. These findings are in accordance with previous studies on gelatin [5] and studies comparing hydrolyzed casein to intact casein [15], where the concentration of most AAs increased significantly after approximately 30 min. The results suggest that an enzymatic hydrolysis is not necessary, if the main goal of consumption is to provide the body with essential AAs (EAAs), BCAAs or any of the less abundant AAs of collagen. As a result of their low content of BCAA and tryptophan, collagen sources have not been anticipated to strongly stimulate muscle protein synthesis. However, a study by Zdzieblik et al. [26] found that ingestion of collagen peptides following exercise led to higher fat free mass (FFM), bone mass (BM), muscle strength and loss of fat mass compared to placebo in elderly sarcopenic men [26]. Taken together with the findings of the present study, it is plausible that an enzymatic hydrolysis is not vital if using collagen as a protein supplement following exercise or for combating sarcopenia. Zdzieblik et al. [26] speculated that the observed benefits of collagen ingestion on muscle protein synthesis could be due to the high content of glycine and its role as a substrate in creatine synthesis [28]. Others have proposed that glycine plays a vital role for collagen’s beneficial effects in relation to OA [22], but also as a metabolite that plays a crucial role in epigenetic regulation and exerts important physiological functions including antioxidative, anti-inflammatory and immunomodulatory effects [29].

NC displayed a significantly higher postprandial plasma concentration of histidine compared to placebo, while EHC did not differ significantly from placebo. This finding could be explained by the fact that the NC product contained a slightly higher amount of histidine (1.1 g/100 g) compared to the EHC product (0.8 g/100 g). NC also displayed a significantly higher postprandial plasma concentration of methionine. This can probably be ascribed to oxidation of methionine to methionine sulfoxide and methionine sulfone taking place during the enzymatic hydrolysis processes, consistent with observations in other mildly processed foods [30,31]. 

Glucose quantification from the ^1^H NMR metabolomics data displayed no significant difference between postprandial glucose concentrations in EHC, NC and placebo. This is in accordance with previous findings reporting that postprandial blood glucose was not significantly affected in healthy individuals following protein ingestion [31,32,33], and other studies comparing postprandial glucose levels following ingestion of intact proteins and their hydrolysate have found similar results. Koopman et al. [15] found no difference in glucose response following consumption of a casein hydrolysate and its intact protein. Similarly, Claessens et al. [34] found no significant differences when investigating postprandial glucose levels following the ingestion of intact soy protein, whey protein and their respective hydrolysates [34]. Thus, an insulin-associated response of protein intake on glucose is probably most strongly associated with differences in the protein content of BCAAs [35].

Proteins receive considerable attention in relation to satiety and high-protein diets have been proposed to be efficient in weight-loss strategies [36]. In association with this, former studies have indicated that collagen-rich AAs may exert unique effects on satiety. Thus, Veldhorst et al. (2009) investigated the effects of different proteins on satiety and subsequent energy intake and found that ad libitum intake was 20% lower after consumption of a breakfast gelatin compared to casein, soy or whey [37]. Similar findings by Hochstenbach-Waelen et al. (2010) reported beneficial short-term effects of gelatin on hunger suppression but no long-term effects on weight maintenance [38]. To the best of our knowledge, these former studies have never been corroborated by studies investigating the effect of collagen processing on satiety potential. We therefore decided to investigate if EHC and NC exerted the same effects on feelings of satiety after intake. Our study revealed no significant differences in feelings of satiety after intake of EHC and NC suggesting that it is merely the AA composition rather than molecular size that determine collagen-derived products’ satiety potential.

## 5. Conclusions

In conclusion, ^1^H NMR-based metabolomics revealed that an enzymatic hydrolysis of collagen was associated with an enhanced absorption rate of glycine, proline and hydroxyproline 20 min post-ingestion. In addition, a greater AUC for glycine, proline and hydroxyproline for an enzymatically hydrolyzed collagen hydrolysate (EHC) suggested a higher bioavailability for EHC compared to a non-enzymatically hydrolyzed collagen hydrolysate (NC). However, an enzymatic hydrolysis did not significantly affect the postprandial absorption rate of other AAs, indicating that an enzymatic hydrolysis only affects absorption and bioavailability of the most abundant AAs of collagen.

## Figures and Tables

**Table 1:** Subject characteristics.

Subjects (*n* = 10)	
Age (y)	26 ± 1
Weight (kg)	77 ± 6
Height (cm)	180 ± 5
Activity level (h) BMI (kg/m^2^)	10 ± 3 24 ± 2

y, years; h, hours of physical activity per week. BMI, body mass index. All values are mean ± SD.

**Table 2:** List of identified metabolites and their chemical shift (δ).

Metabolite	δ ^1^H (multiplicity)
Alanine	1.46 (d), 3.76 (q)
Arginine	1.68 (m), 1.90 (m), 3.23 (t), 3.76 (t)
Asparagine	4.00 (dd), 2.94 (m), 2.84 (m)
Glucose	3.233 (dd), 3.398 (m), 3.458 (m), 3.524 (dd), 3.726 (m), 3.824 (m), 3.889 (dd), 4.634 (d), 5.223 (d)
Glycine	3.54 (s)
Histidine	3.16 (dd), 3.23 (dd), 3.98 (dd), 7.09 (d), 7.90 (d)
Isoleucine	0.926 (t), 0.997 (d), 1.246 (m), 1.475 (m), 1.968 (m), 3.661 (d)
Leucine	0.948 (t), 1.700 (m), 3.722 (m)
Lysine	1.46 (m), 1.71 (m), 1.89 (m), 3.02 (t), 3.74 (t)
Methionine	2.157 (m), 2.631 (t), 3.851 (dd)
Phenylalanine	3.19 (m), 3.98 (dd), 7.32 (d), 7.36 (m), 7.42 (m)
Proline	1.99 (m), 2.06 (m), 2.34 (m), 3.33 (dt), 3.41 (dt), 4.12 (dd)
Serine	3.832 (dd), 3.958 (m)
Threonine	1.316 (d), 3.575 (d), 4.244 (m)
Tyrosine	3.024 (dd), 3.170 (dd), 3.921 (dd), 6.877 (m), 7.170 (m)
Valine	0.976 (d), 1.029 (d), 2.261 (m), 3.691 (d)
Hydroxyproline	2.14 (ddd), 2.42 (M), 3.36 (ddd), 3.46 (dd), 4.33 (d), 4.35 (d)

s = singlet, d = doublet, dd = doublet of doublets, ddd = doublet of doublet of doublets, dt = doublet of triplets, m = multiplet, t = triplet, q = quartet.

**Table 3:** Pairwise comparisons of dietary treatments for all amino acids (AAs).

Comparison	EHC vs. NC			EHC vs. Placebo			NC vs. Placebo		
AA	EHC	NC	*p*	EHC	Pl	*p*	NC	Pl	*p*
Alanine	367.4	351.3	0.69	367.4	266.2	0.0002	351.3	266.2	0.001
Arginine	86.54	82.54	0.67	86.54	50.28	<0.0001	82.54	50.28	<0.0001
Asparagine	40.60	40.43	0.99	40.60	34.58	0.019	40.43	34.58	0.023
Glutamate	39.17	34.34	0.25	39.17	25.68	0.0006	34.34	25.68	0.021
Glutamine	513.5	521.9	0.87	513.5	469.2	0.043	521.9	469.2	0.015
Glycine	448.7	380.7	0.0059	448.7	204.1	<0.0001	380.7	204.1	<0.0001
Histidine	72.85	77.37	0.12	72.85	67.38	0.053	77.37	67.38	0.0006
Hydroxyproline	64.92	48.88	0.0087	64.92	1.484	<0.0001	48.88	1.484	<0.0001
Isoleucine	73.62	68.00	0.21	73.62	58.24	0.0003	68.00	58.24	0.017
Leucine	127.4	120.8	0.42	127.4	99.09	<0.0001	120.8	99.09	0.0014
Lysine	122.5	119.1	0.79	122.5	99.37	0.0008	119.1	99.37	0.0034
Methionine	18.78	22.51	0.034	18.78	17.42	0.59	22.51	17.42	0.0043
Proline	335.4	285.8	0.0074	335.4	186.3	<0.0001	285.8	186.3	<0.0001
Phenylalanine	47.65	48.21	0.93	47.65	39.80	0.0003	48.21	39.80	0.0001
Serine	147.7	145.0	0.89	147.7	108.5	<.0001	145.0	108.5	<0.0001
Threonine	152.2	148.2	0.83	152.2	120.6	0.0006	148.2	120.6	0.0023
Tyrosine	56.47	55.91	0.96	56.47	48.72	0.011	55.91	48.72	0.018
Valine	278.4	254.9	0.09	278.4	213.2	<0.0001	254.9	213.2	0.0027
BCAA	479.5	443.7	0.15	479.5	370.6	<0.0001	443.7	370.6	0.002
Total AA	2994	2806	0.14	2994	2110	<0.0001	2806	2110	<0.0001
EAA	893.4	859.1	0.48	893.4	715.2	<0.0001	859.1	715.2	0.0003
Gly-Pro-Hp	849.1	715.4	0.0003	849.1	391.8	<0.0001	715.4	391.8	<0.0001

EHC, enzymatically hydrolyzed collagen; NC, non-enzymatically hydrolyzed collagen; Pl, placebo; BCAA, Branched chain amino acids; AA, amino acids; EAA, essential AA; Gly-Pro-Hp, sum of glycine, proline and hydroxyproline. Data are mean concentrations (μM). *p* = statistical *p*-value.

## Supplementary Materials

The following are available online at https://www.mdpi.com/2072-6643/11/5/1064/s1, Table S1. Amino acid composition of the two collagen products included in the study, Figure S1: Plasma concentration of methionine for EHC, NC and placebo, Figure S2. Plasma concentrations of alanine, asparagine, glutamate, glutamine, histidine, serine, threonine and valine over time following ingestion of EHC, NC and placebo, Figure S3. Plasma concentrations of arginine, leucine, isoleucine, lysine, phenylalanine and total AAs over time following ingestion of EHC, NC and placebo, Figure S4. Plasma concentrations of EAA and BCAA over time following ingestion of EHC, NC and placebo, Figure S5. (A) Plasma concentrations of glucose over time for EHC, NC and placebo. (B) AUC is evaluated as incremental AUC from time −20 min (Pre) to 240 min, Figure S6. Effects of EHC, NC and placebo on feelings of hunger over time.
