# Supplementary material for: Enzymatic Hydrolysis of a Collagen Hydrolysate Enhances Postprandial Absorption Rate—A Randomized Controlled Trial
Source: Nutrients. 2019 May 13;11(5):1064. doi: 10.3390/nu11051064 (PMC6566347; doi:10.3390/nu11051064)
Supplement: Supplementary file 1 [file nutrients-11-01064-s001.pdf]

Supplementary material

Table S1. Amino acid composition of the two collagen products included in the study

**Amino acid**	**Non-enzymatically hydrolyzed product (NC) (g/100 g product)**	**Enzymatically hydrolyzed product (EHC) (g/100 g product)**
Alanine	8.8	8.7
Arginine	7.5	7.3
Aspartic acid	6.0	6.1
Cysteine	<1.0	<1.0
Glutamic acid	11.0	10.0
Glycine	20.0	21.6
Histidine	1.1	0.81
Hydroxyproline	10.2	10.6
Isoleucine	1.7	1.5
Leucine	3.7	3.4
Lysine	3.8	3.6
Methionine	0.9	0.9
Phenylalanine	2.3	2.2
Proline	12.0	11.6
Serine	3.4	3.2
Threonine	2.1	2.1
Tryptophan	0.36	0.08
Tyrosine	1.3	0.9
Valine	3.0	2.5

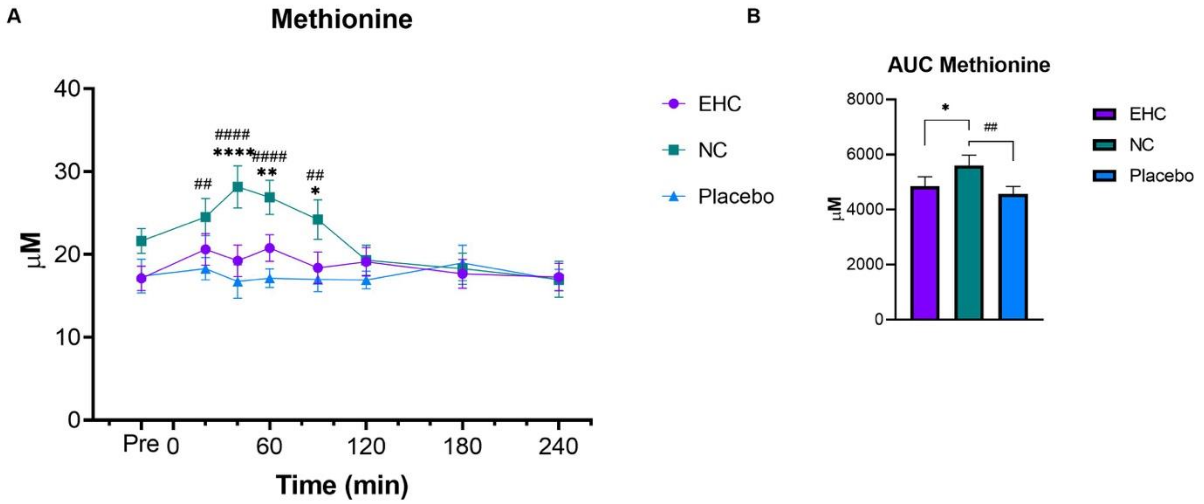

Figure S1. *Plasma concentration of methionine for EHC, NC and placebo. Time for beverage consumption is designated time zero (0 min) and first blood sample was drawn 20 min prior to consumption (Pre).* ***A)*** *Concentration of methionine over time for each of the three intervention beverages.* ***B)*** *Area under the curve (AUC) analysis evaluated from time = -20 min. All data are mean ± SEM. * Significant differences between EHC and NC. # significant differences between either collagen products and placebo. * p<0.05, ** p<0.002 *** p<0.0002, **** p<0.0001, # p<0.05, ## p<0.002 ### p<0.0002, #### p<0.0001*

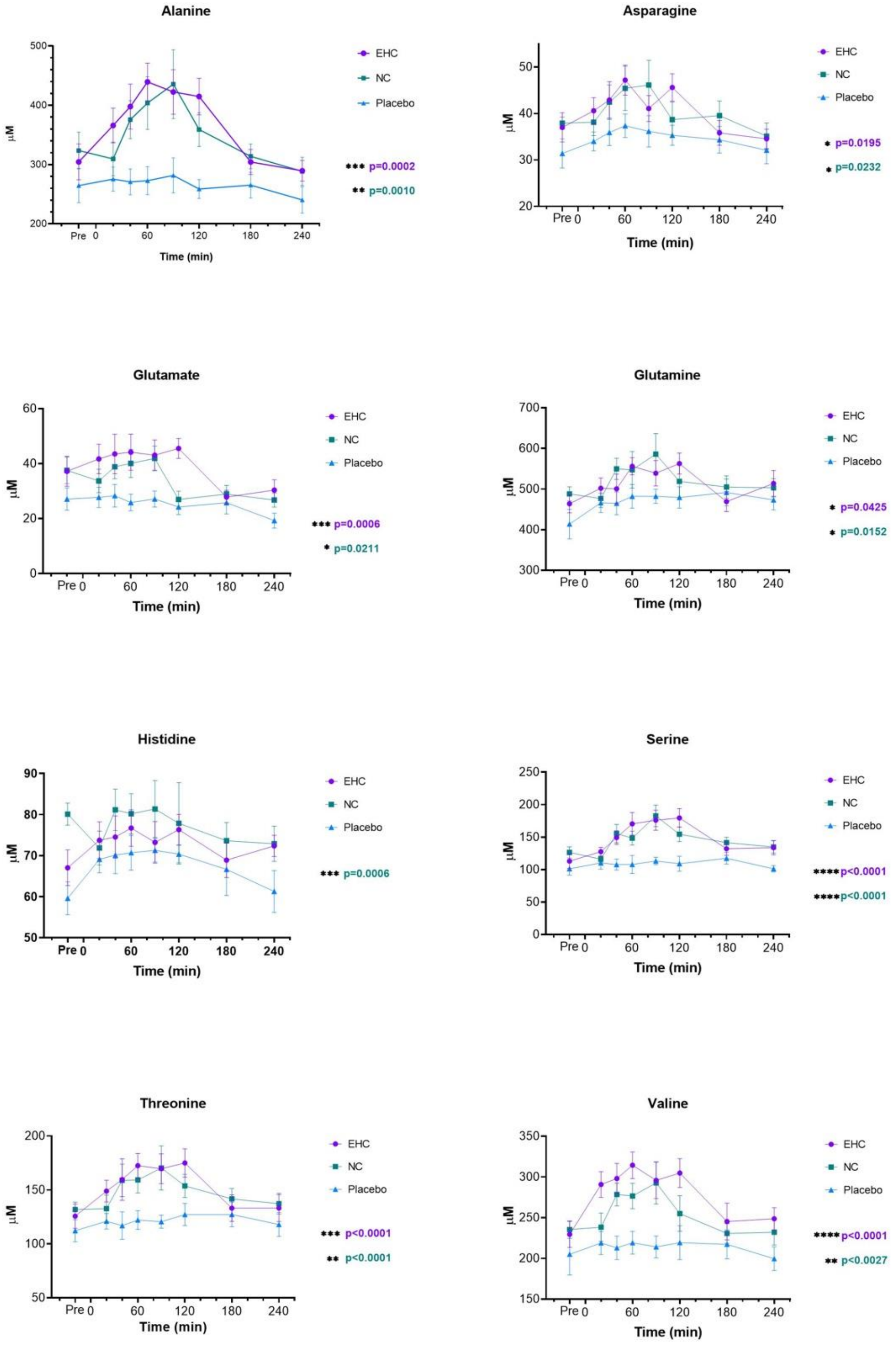

Figure S2. *Plasma concentrations of alanine, asparagine, glutamate, glutamine, histidine, serine, threonine and valine over time following ingestion of EHC, NC and placebo. Time for beverage consumption is designated time zero (0 min) and first blood sample was drawn 20 min prior to consumption (Pre). A significant difference was observed between either collagen supplements and placebo, but not between the two collagen supplements. Colored p-values indicate significant difference between corresponding collagen supplement (purple = EHC, green = NC) and placebo.*

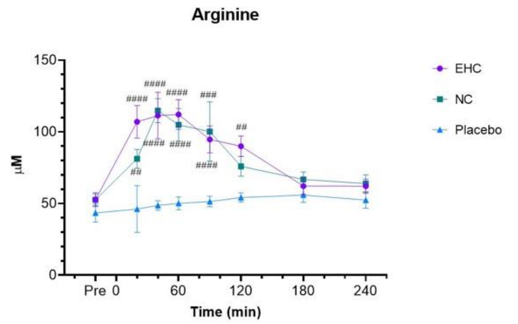

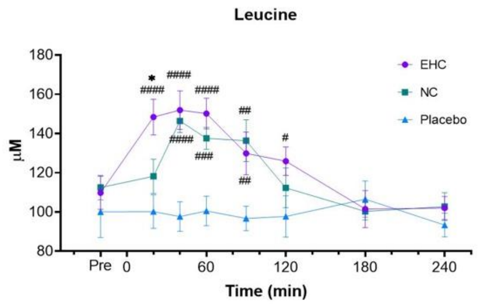

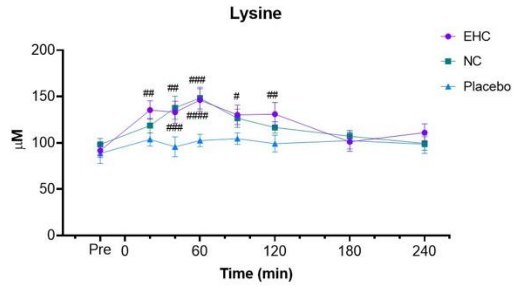

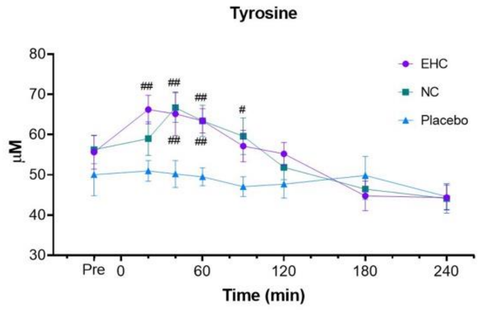

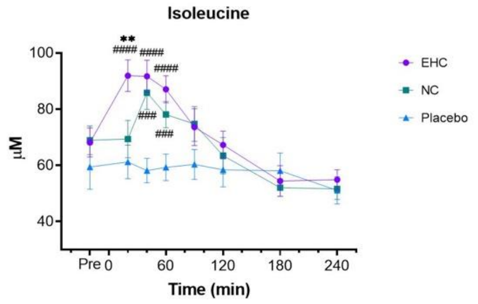

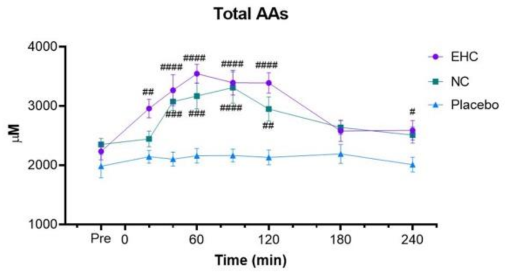

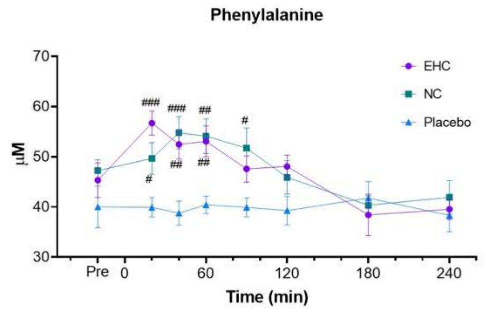

Figure S3. *Plasma concentrations of arginine, leucine, isoleucine, lysine, phenylalanine and total AAs over time following ingestion of EHC, NC and placebo. Time for beverage consumption is designated time zero (0 min) and first blood sample was drawn 20 min prior to consumption (Pre). * Significant time*treatment interactions between EHC and NC. # significant time*treatment interactions between either collagen products and placebo. All data are mean ± SEM. * p<0.05, ** p<0.002, # p<0.05, ## p<0.002, ### p<0.0002, #### p<0.0001*

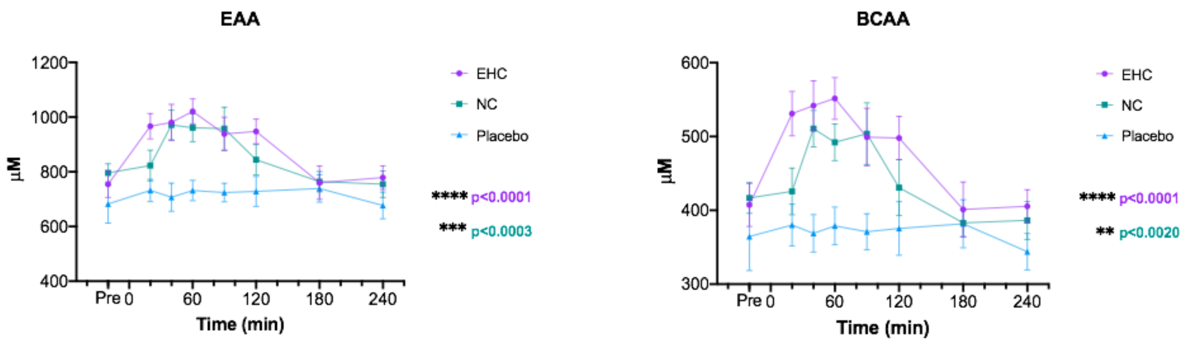

Figure S4. *Plasma concentration of EAA and BCAA over time following ingestion of EHC, NC and placebo. Time for beverage consumption is designated time zero (0 min) and first blood sample was drawn 20 min prior to consumption (Pre). A significant effect was observed between either collagen supplements and placebo, but not between the two collagen supplements. Colored p-values indicate significant difference between corresponding collagen supplement (purple = EHC, green = NC) and placebo.*

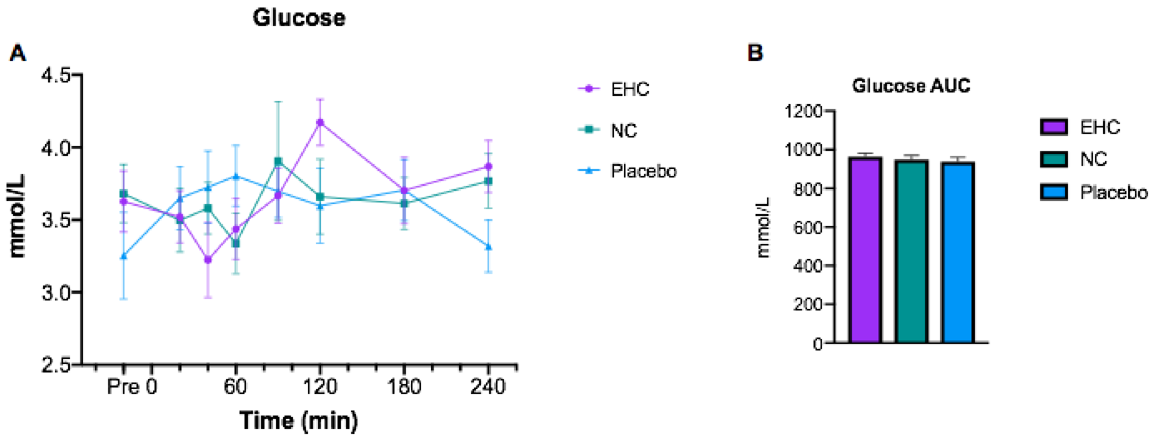

Figure S5. *A) Plasma concentration of glucose over time for EHC, NC and placebo. Time for beverage consumption is designated time zero (0 min) and first blood sample was drawn 20 min prior to consumption (Pre).* ***B)*** *AUC is evaluated as incremental AUC from time -20 min (Pre) to 240 minutes. No significant difference was observed in plasma concentration of glucose or AUC for any of the three dietary treatments. All data are mean ± SEM.*

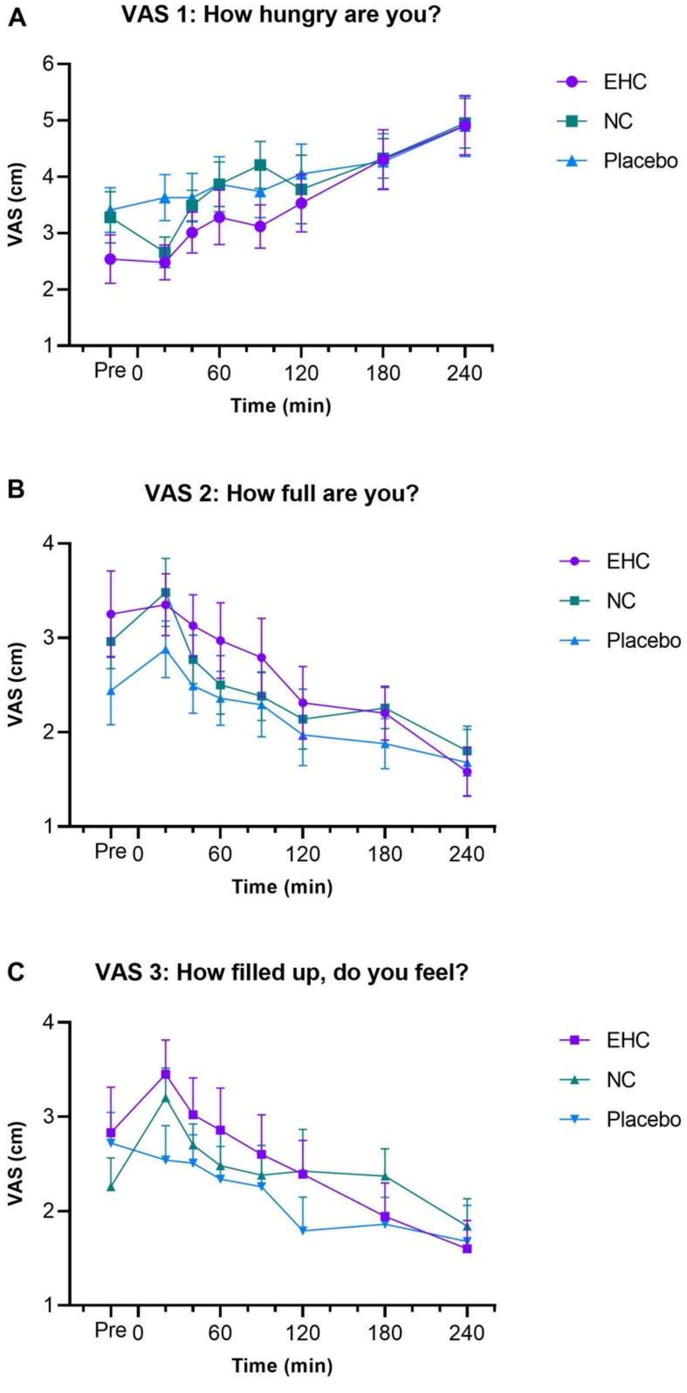

Figure S6. *Effect of EHC, NC and placebo on feelings of hunger over time. Time for beverage consumption is designated time zero (0 min) and first blood sample was drawn 20 min prior to consumption (Pre).* ***A)*** *Mean VAS 1 score over time for EHC, NC and placebo.* ***B)*** *Mean VAS 2 score*

over time for EHC, NC and placebo. ***C****) Mean VAS 3 score over time for EHC, NC and placebo. Values are mean ± SEM.*
